# Agroecological Advantages of Early-Sown Winter Wheat in Semi-Arid Environments: A Comparative Case Study From Southern Australia and Pacific Northwest United States

**DOI:** 10.3389/fpls.2020.00568

**Published:** 2020-05-27

**Authors:** David J. Cann, William F. Schillinger, James R. Hunt, Kenton D. Porker, Felicity A. J. Harris

**Affiliations:** ^1^Department of Animal, Plant and Soil Sciences, La Trobe University, Melbourne, VIC, Australia; ^2^Department of Crop and Soil Sciences, Washington State University, Dryland Research Station, Lind, WA, United States; ^3^Crop Sciences, Agronomy Group, South Australian Research and Development Institute, Urrbrae, SA, Australia; ^4^School of Agriculture, Food & Wine, Waite Research Institute, The University of Adelaide, Urrbrae, SA, Australia; ^5^NSW Department of Primary Industries, Wagga Wagga Agricultural Institute, Wagga Wagga, NSW, Australia

**Keywords:** winter wheat, climate change, adaptation, vernalization, deep sowing, yield gap, drought

## Abstract

Wheat (*Triticum aestivum* L.) is the most widely-grown crop in the Mediterranean semi-arid (150–400 mm) cropping zones of both southern Australia and the inland Pacific Northwest (PNW) of the United States of America (United States). Low precipitation, low winter temperatures and heat and drought conditions during late spring and summer limit wheat yields in both regions. Due to rising temperatures, reduced autumn rainfall and increased frost risk in southern Australia since 1990, cropping conditions in these two environments have grown increasingly similar. This presents the opportunity for southern Australian growers to learn from the experiences of their PNW counterparts. Wheat cultivars with an obligate vernalization requirement (winter wheat), are an integral part of semi-arid PNW cropping systems, but in Australia are most frequently grown in cool or cold temperate cropping zones that receive high rainfall (>500 mm p.a.). It has recently been shown that early-sown winter wheat cultivars can increase water-limited potential yield in semi-arid southern Australia, in the face of decreasing autumn rainfall. Despite this research, there has to date been little breeding effort invested in winter wheat for growers in semi-arid southern Australia, and agronomic research into the management of early-sown winter wheat has only occurred in recent years. This paper explores the current and emerging environmental constraints of cropping in semi-arid southern Australia and, using the genotype × management strategies developed over 120 years of winter wheat agronomy in the PNW, highlights the potential advantages early-sown winter wheat offers growers in low-rainfall environments. The increased biomass, stable flowering time and late-summer establishment opportunities offered by winter wheat genotypes ensure they achieve higher yields in the PNW compared to later-sown spring wheat. Traits that make winter wheat advantageous in the PNW may also contribute to increased yield when grown in semi-arid southern Australia. This paper investigates which specific traits present in winter wheat genotypes give them an advantage in semi-arid cropping environments, which management practices best exploit this advantage, and what potential improvements can be made to cultivars for semi-arid southern Australia based on the history of winter wheat crop growth in the semi-arid Pacific Northwest.

## Introduction

The cropping systems of Mediterranean semi-arid southern Australia are diverse, resilient and responsive to change. Bread wheat (*Triticum aestivum* L.) is the most widely grown crop in the region and is important for both local consumption and export. The effects of anthropogenic climate change, including rising maximum temperatures, decreasing minimum temperatures and growing season rainfall and a delayed onset of autumn rain sufficient for germination (referred to as the “autumn break” or “breaking rain”) present new challenges to which growers will need to respond in order to maintain farm yields and profitability (Pook et al., 2009; Cai et al., 2012; Crimp et al., 2016b; Hochman et al., 2017). Within a context of rising business costs and increased income risk these effects have already been visible across southern Australia since 1990, decreasing water-limited potential wheat yield (PY_W_) and increasing the difficulty of establishing crops during autumn, the traditional sowing period for spring wheat (Pook et al., 2009; Hochman et al., 2017). A resilient response to the challenges of a changing environment is unlikely to be achieved through a singular technical or genetic development. Historic increases in Australian wheat yields have not been the result of individual advancements in crop genotype or crop management, but instead when the combination of crop genotype, environment and management has created synergies through which yield is increased more than can be accounted for through each development alone (Kirkegaard and Hunt, 2010; Hunt et al., 2019a).

The effects of anthropogenic climate change on southern Australia, both visible and predicted (including reduced growing season rainfall and establishment opportunities during autumn), have caused cropping conditions in semi-arid regions to converge with those of the inland Pacific Northwest (PNW) of the United States. For over a century, growers in the PNW have successfully grown wheat in drier conditions than those of semi-arid southern Australia, including in the absence of sufficient late summer and autumn rainfall to establish crops. This paper argues that although differences exist between the environmental conditions and production systems of the two regions, there is a benefit in applying some of the genotype × management synergies that have been successful in the low-rainfall regions of the PNW to wheat production in semi-arid southern Australia.

This is particularly the case for winter wheat, which has long been grown in the PNW, but only recently become of interest to growers in low-rainfall Australian environments, who more frequently grow spring wheat (Hunt, 2017). Winter wheat is differentiated from spring wheat by an obligate vernalization requirement, meaning that it must accumulate sufficient vernal time when temperatures are cool (−1.3 to 15.7°C, Porter and Gawith, 1999) in order to begin reproductive development. Many of the synergies used by PNW growers to increase yield have been facilitated by advantages winter wheat offers over spring wheat, and also have potential benefits for southern Australian growers.

This paper compares semi-arid cropping conditions in southern Australia and the inland PNW and demonstrates the confluence between the two environments. Focusing on the prevailing and emerging constraints inherent in the crop production environments (E) of both regions, we then discuss how specific genotype (G) × management (M) synergies offered by winter wheat cultivars are used to overcome each of these constraints in the PNW, and how they could similarly be employed in southern Australia. Finally, we consider barriers that may prevent implementation of early-sown winter wheat in southern Australia, and the conditions that would need to be met for widespread adoption of the practice.

## Inland Pacific Northwest

### Cropping Environments

**FIGURE 1:**
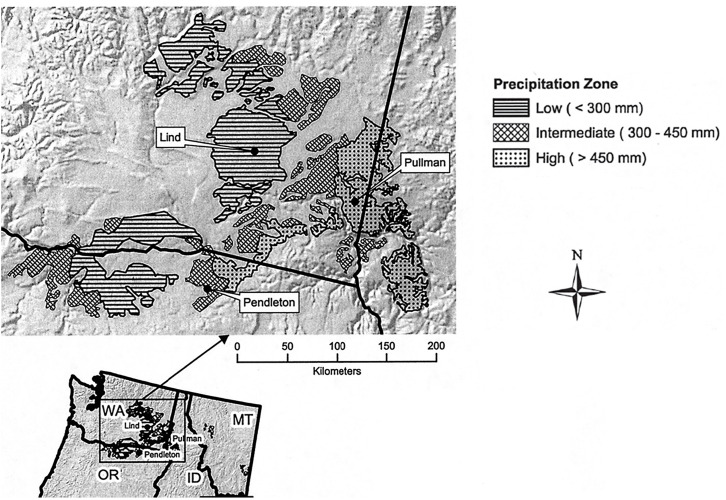
Map showing the low (<300 mm annual precipitation), intermediate (300–450 mm) and high (>450 mm) precipitation zones of the inland PNW. Reprinted from Schillinger et al. (2006) with permission.

**FIGURE 2:**
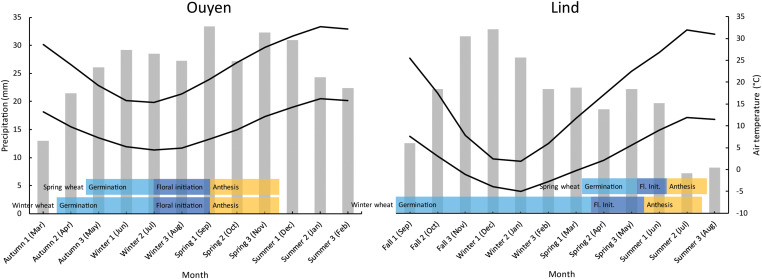
Comparison of seasonal temperatures and precipitation for Ouyen in southern Australia and Lind, WA in the PNW, and seasonal fit of winter wheat and spring wheat life cycles under current management.

The inland PNW is commonly divided into three annual precipitation zones: (i) low, <300 mm of precipitation; (ii) intermediate, 300–450 mm of precipitation, and; (iii) high, 450–600 mm of precipitation (Figure 1). Dryland wheat is grown on approximately 3,350,000 hectares in the inland PNW of which 1,557,000 hectares is in the low-precipitation zone (Schillinger et al., 2006). The low-precipitation zone, where early sowing of winter wheat is practiced, is most comparable to semi-arid southern Australia and is therefore the focus of the PNW portion of this paper. Precipitation intensities and volumes are low, usually not exceeding 2–3 mm/h and 10–20 mm per event. About 70% of annual precipitation occurs from October through March and 25% from April through June. July through September is the driest period (Figure 2). The Mediterranean-like climate of the inland PNW is largely influenced by frontal weather systems moving with prevailing westerly winds from the Pacific Ocean. The Cascade Mountains to the west impose a rain shadow effect. The driest part of the inland PNW is just east of the Cascade Mountains where average annual precipitation drops to 125 mm and gradually rises west to east with increase in elevation to 600 mm in the Palouse region.

Winter weather is cool to cold with mean daily temperature in December and January of −1 and −2°C, respectively, but occasionally dropping to −24°C or lower. During extreme cold, soil not covered with snow may freeze to depths of 40 cm which can lead to heavy water runoff and soil erosion when weather changes bring rain or cause rapid snow melt. During summer, high-pressure systems dominate, leading to warm, dry conditions and low relative humidity. Average maximum temperatures in summer range between 20 and 35°C.

In the PNW, anthropogenic climate change has resulted in warming of air temperatures, however, no shifts in precipitation amounts have yet been documented (Schillinger and Papendick, 2008; Karimi et al., 2017). Climate models predict increases in winter precipitation but drier summers in the PNW. Combined with elevated CO_2_, which promotes crop growth and improves its transpiration efficiency, wheat grain yield potential in the PNW is predicted to increase (Stöckle et al., 2018), despite the otherwise dire global environmental consequences of such climate change.

### Wheat Production

Winter wheat has been grown in PNW since the early 1890s. Before then, growers planted spring wheat annually (i.e., only a short 7-month fallow between crops) and grain yields were highly variable. Beginning in 1890, cold-hardy wheat cultivars brought in by rail from the eastern United States were planted and survived the winters but were not widely adopted by growers due to severe shattering loss before grain harvest. However, in 1896, growers experimenting with Jones Winter Fife, a soft red cultivar bred in New York and brought in by train, found this cultivar could both survive the winter and reach maturity with minimal shattering. Growers were soon convinced that the best grain yields could be achieved by planting winter wheat (Schillinger and Papendick, 2008). Soon thereafter, seed of Turkey Red, a hard-red winter wheat cultivar developed in the Crimea, was introduced to the region by homesteading immigrants arriving to the PNW from the United States Great Plains. Today, the major class of wheat produced is soft white with 90% exported to overseas markets from ship loading facilities in Portland, Oregon where it is used to make cakes, noodles, flatbreads, breakfast cereal, pastries and other products (Washington Grain Commission, 2019).

Winter wheat in the PNW drylands is planted in late summer, fulfils its vernalization requirement during winter before entering reproductive growth in spring and then matures in summer (Figure 2). Growers are generally hesitant to plant any spring crop because yields are highly variable and are not nearly as economically viable as winter wheat. For example, continuously cropped spring wheat (i.e., one crop per year and no 13-month fallow) at Lind, WA (244 mm annual precipitation) consistently produces less than 40% of the grain yield of winter wheat after a 13-month fallow (Schillinger, 2016) and at Ritzville, WA (292 mm p.a.) 55% of the grain yield of winter wheat after fallow (W. F. Schillinger, unpublished). Several studies have shown that late-sown winter wheat and spring wheat (as well as other spring-sown crops), by a wide margin, are not economically competitive with early-sown winter wheat in the drylands of east-central Washington (Juergens et al., 2004; Schillinger et al., 2007; Bewick et al., 2008).

Dryland wheat farming is practiced in areas of south-central Washington that receive as little as 150 mm average annual precipitation; this is considered the lowest for dryland wheat production in the world. A 2-year rotation of winter wheat followed by a 13-month fallow (only one crop every 2 years) is widely practiced in the low-precipitation zone. Experience to date shows that all winter crops in the drylands require a preceding 13-month fallow period so that they may be sown into stored moisture in late August-early September to produce an economically viable yield. Average winter wheat (one crop every other year) yields range from 1.3 to 4.4 t/ha with 150 and 300 mm of annual precipitation, respectively. Wheat grain yields have continued to increase linearly since the 1950s due to advances in breeding and genetics, modern farm equipment, and agronomic practices. In the past 10 years, growers in the low-precipitation zone have begun planting some acreage to winter pea (*Pisum sativum* L.), winter canola (*Brassica napus* L.), and winter triticale (× *Triticosecale Wittmack*). Details on dryland farming throughout the PNW are found in Schillinger et al. (2006) and possible future shifts in PNW cropping systems in response to climate change are outlined in Karimi et al. (2017) and Stöckle et al. (2018). Tables 1–3 summarize the environmental, genotypic and management contexts of the low-precipitation PNW, and compare them to conditions in semi-arid southern Australia.

**TABLE 1:** Comparative analysis of case studies: environmental conditions.

Factor	Inland pacific northwest	Semi-arid southern Australia
Annual rainfall	Low rainfall zone: <300 mm Intermediate RZ: 300–450 mm High RZ: 450–600 mm	280–400 mm
Growing season length	Winter wheat: 11 months (September–July) Spring wheat: 6 months (April–August)	Winter wheat: 8 months (April–November) Spring wheat: 7 months (May–November)
Optimal flowering period	Late spring (May)	Early spring (September)

**TABLE 2:** Comparative analysis of case studies: genotypic conditions.

Factor	Inland pacific northwest	Semi-arid southern Australia
Vernalization requirement of common wheat cultivars	Obligate vernalization requirement (winter wheat)	No obligate vernalization requirement (spring wheat)
Coleoptile length	<90 mm	<70 mm
Number of suitable winter wheat cultivars available	>20	3 (released 2016–2019)
Grain quality and products	Soft white (cakes, noodles etc.)	Hard white (bread and noodles)

**TABLE 3:** Comparative analysis of case studies: management conditions.

Factor	Inland pacific northwest	Semi-arid southern Australia
Sowing time	Winter wheat: late summer (August–September) Spring wheat: late winter-early spring (March–April)	Winter wheat: early autumn (March–April) Spring wheat: late autumn (May)
Driver of winter wheat emergence	Stored soil moisture	Rainfall
Common rotation with wheat	Long fallow (13 months)	Barley, canola, pulse crops, long fallow (17 months)
Winter wheat sowing depth	100–180 mm	20–50 mm

## Semi-Arid Southern Australia

### Cropping Conditions

**FIGURE 3:**
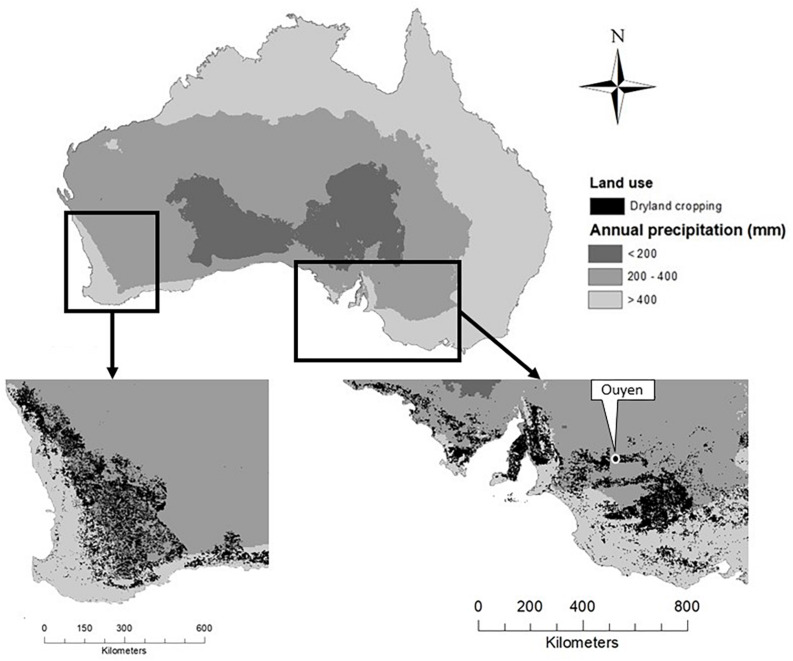
Map of Australia showing semi-arid regions (200–400 mm annual precipitation; mid-gray) and dryland cropping sites in southern Australia (black).

Rainfall distribution in southern Australia is Mediterranean, with wet winters (June–August) and dry summers (December–February). Annual precipitation in cropping regions ranges from 281 mm at Waikerie on the dry fringe of the low rainfall zone to 747 mm at Millicent in the high rainfall zone (BOM, 2019a, c). Cropping in low-precipitation (<400 mm p.a.) environments occurs in central and south-western New South Wales, the Mallee districts of north-western Victoria and eastern South Australia, the Upper North and Eyre Peninsula of South Australia and much of south-western Western Australia (Figure 3). Periods of low rainfall during summer are accompanied by high temperatures (Figure 2). Annual precipitation in these low-precipitation regions is highly variable seasonally and across locations, ranging from 179 mm in 2012 to 573 mm in 2010 at Ouyen, Victoria (BOM, 2019b).

Since 1990, temperatures in southern Australian have increased, and growing season rainfall decreased by 11%; both phenomena have been linked to anthropogenic climate change (Murphy and Timbal, 2008; BOM and CSIRO, 2018). Much of this decline in rainfall has occurred during autumn (March–May). These trends have been attributed to a poleward expansion of the subtropical dry zone (Cai et al., 2012) and declining frequency of cold-cored, cut-off low synoptic systems (Pook et al., 2009; Cai et al., 2012). Autumn-breaking rains, used to establish crops, are also occurring later. Pook et al. (2009) demonstrated that the number of days until an “ideal” break occurs has increased by 6.3 days per decade since the 1890s. These factors have contributed to an estimated 27% decrease in water-limited potential yield across southern Australia since 1990 (Hochman et al., 2017), bringing potential yield closer to yields seen in the PNW. While advances in crop agronomy and plant breeding have prevented on-farm yields from declining over the same period, further advancements will be required to maintain or improve both actual yields and water-limited potential yield in the future.

### Wheat Production

Cereal production is the most common land use in semi-arid cropping regions of southern Australia, with wheat the primary crop, followed by barley (*Hordeum vulgare* L.). Cereal crops are grown in rotation with non-cereal crops, with canola being the most common (Collins and Norton, 2019). Common pulse crops include chickpea (*Cicer arietinum* L.), field (dry) pea, lentil (*Lens culinaris*) and narrow-leaf lupins (*Lupinus angustifolius*). As the growing season is shorter in southern Australia than the PNW (Figure 2), a long fallow (where a field is out of crop for one whole growing season) lasts 16–18 months, compared to 13 months in the PNW. The widespread availability of crop species suitable to rotate with wheat means that long fallows are less common on modern southern Australian farms compared to their PNW counterparts.

In southern Australia, the British colonialists of the late 18th century attempted to grow winter wheat but found their life cycle poorly matched seasonal conditions in semi-arid regions, and production was restricted to the few high rainfall regions with soils and terrain suitable for crop production. Spring wheat cultivars have been the mainstay of Australian wheat production since the beginning of the 20th century, and their development at this time revolutionized the industry and allowed reliable wheat production in semi-arid areas (Pugsley, 1983).

With a relatively mild winter, spring wheat can be sown in late autumn or early winter, begin reproductive development at the end of winter and mature in early summer (Figure 2). This contrasts to the PNW where winters are cold enough to kill spring wheat in tillering or early stages of reproductive development, and spring wheat must be planted in spring. However, although temperatures in Australia have been on a warming trend since the 1960s, incidences of frost, particularly late in the growing season, have increased in parts of southern Australia (Crimp et al., 2016a). The change has been most pronounced in semi-arid cropping regions of southern Australia, where simulation shows yield reductions due to frost were 20–60% higher from 1986–2013 compared to 1960–1985 (Crimp et al., 2016b). Environmental conditions during winter are therefore trending toward those found in the PNW, albeit less severely.

Crop establishment relies on the ‘autumn break’, which is the first significant rainfall event of the growing season, and has been variously defined as either 25 mm of rainfall within a 3-day period, or 30 mm over 7 days (Pook et al., 2009). A more mechanistic definition is provided by Unkovich (2010) who described it the first week in autumn where precipitation exceeds pan evaporation. A wheat crop may be sown following these rains in late autumn or early winter (late April–June; Figure 2), or ‘dry sown’ ahead of rainfall to ensure timely establishment once sufficient rainfall to induce germination has occurred (Fletcher et al., 2015). Flowering occurs during early spring (September) and harvest in early summer (November–December). The hot, dry summers of semi-arid southern Australia prevent any crop growth from December to March. Under best practice, fields are kept free of growing plants from the harvest of one crop until sowing the following year to maximize accumulation of water from irregular rainfall events for subsequent crop use (Hunt et al., 2013).

In recent years, there has been revived interest in winter wheat amongst growers, researchers and breeders in the semi-arid regions of southern Australia (Hunt, 2017). Firstly, the decline in autumn rainfall described above has coincided with the optimal timing of establishment for the popular fast developing spring wheat cultivars. Secondly, no-till farming has allowed sowing time to move earlier (by 30 days over 3 decades, Anderson et al., 2016) to the point where sowing times for fast spring cultivars are optimal for the first time (Flohr et al., 2018). For growers to sow any earlier, cultivars that are slower to develop through their life cycle are required (Hunt et al., 2019b). Incentive for growers to continue the trend for earlier sowing has been driven by a continual increase in farm size and cropping intensity (Fletcher et al., 2016), and in conjunction with winter wheat, can increase water limited yield potential by ∼15% compared to later sown spring wheat (Hunt, 2017). However, breeding companies in Australia have only recently responded to the demand for winter wheat in semi-arid regions (Table 2).

Changes in autumn precipitation patterns and grower shifts toward early sowing have caused the farming systems of southern Australia to converge with those of PNW. One potential lesson would be a deeper understanding of the role that early sown winter wheat plays in PNW production systems, and how improved breeding of winter cultivars and optimized management can lead to increased yield and manage environmental constraints. There are several emerging environmental challenges that Australian growers will need to overcome in order to increase on-farm yield. These challenges will not have simple, one-dimensional solutions and overcoming them will require the synergy of genotypic and management strategies (Table 4).

**TABLE 4:** Current and emerging environmental constraints (E) to wheat production in Mediterranean semi-arid environments and the corresponding genotype (G) and management (M) advantages offered by winter wheat cultivars.

Environmental constraint (E)	Genotypic advantage of winter wheat (G)	Management strategies to maximize winter wheat yield advantage (M)
Low water-limited potential yield	Increased biomass Increased rooting depth	Early sowing
Few establishment opportunities in autumn		Deep sowing Long fallow
Reproductive frost risk	Winter hardiness	
Narrow optimal flowering period	Stable flowering time Winter hardiness	Early sowing
Increasing business costs and production risk	Stable flowering time	Grazing Whole-farm timeliness

## Narrow Optimal Flowering Periods in Mediterranean Semi-Arid Environments (E)

A major determinant of wheat yield in water-limited environments is flowering time (Fischer et al., 1990; Gomez-Macpherson and Richards, 1995; Bodner et al., 2015). In the Australian wheat belt, low radiation, cold temperatures and frost during winter, as well as hot, dry conditions in late spring and summer, define a period of least harm, known as the optimal flowering period (OFP; Flohr et al., 2017). During the OFP, the risk of yield reductions caused by frost, heat, or drought is balanced and, on average, yield damage is minimized. Less work has been done defining an OFP for growing regions in the PNW, but there are certain yield penalties for wheat that encounters freezing air temperatures during flowering in mid-to-late May or has not finished reproductive growth by the onset of hot, dry weather in late June (Gizaw et al., 2018).

In temperate regions, OFPs tend to be broad, meaning wheat can flower across a wider range of dates and still maximize yield. However, the southern Australian growing season is immediately succeeded by a distinct hot, dry season, creating a much narrower OFP than more temperate climates. The importance of timely flowering is therefore more heavily weighted in semi-arid Mediterranean regions such as those found in the southern Australian wheat belt.

While OFPs have perhaps not been as well defined for the PNW, the period of least harm for flowering crops is similarly narrow, as winter and early spring weather conditions are harsher than those found in southern Australia, and frosts often occur in draws and other low-lying areas in mid-to-late May during the booting and flowering stages of early-sown winter wheat with resulting severe decline in grain yield (Donaldson, 1996). The risk of heat and drought conditions in late spring and early summer during grain development is similarly high to that in southern Australia (Figure 2).

### Stable Flowering Time (G)

In Australia, early sown winter wheat flowering time is stable relative to spring wheat across a range of sowing dates (Flohr et al., 2018). The European winter wheat cultivars brought to Australia by British colonists flowered much later than spring wheat established in late autumn, and were more likely to suffer heat and drought damage than spring wheat. However, modern winter wheat bred under Australian conditions, when established early, can develop fast enough to flower at a similar time as spring wheat sown later, and thus there is no increased risk of drought, heat and frost damage compared to spring wheat (Flohr et al., 2018). This has been achieved by shortened crop lifecycle due to reduced vernalization requirement and photoperiod sensitivity.

Winter wheat in semi-arid southern Australia therefore offers an advantage in environments with narrow OFPs. Winter wheat and spring wheat planting opportunities overlap in Australia. In seasons when soil moisture is sufficient for establishment prior to the traditional sowing period (late autumn), sowing spring wheat is untenable due to the high risk of early flowering, frost damage and reduced yield potential. In contrast, sowing of a winter wheat cultivar enables early establishment and timely flowering. Winter wheat established early generally yields more than spring wheat established late, particularly in years where the soil profile is filled with water and root growth is optimal (Coventry et al., 1993; Penrose, 1993). Sowing winter wheat therefore not only increases flowering time stability, but also yield stability (Flohr et al., 2018). The flowering time stability of winter wheat also operates across seasons, reducing yield penalties associated with chronically high air temperatures and accelerated development (Hunt et al., 2018).

Cold temperatures during winter in the PNW ensure that winter wheat always fulfils its vernalization requirement. While freezing temperatures are still common during early spring, risk of frost has diminished considerably by mid-May and booting and flowering during this period balances the risk of frost with the risk of heat and drought damage, which is common in late spring and early summer. Early-sown winter wheat in the PNW will always flower earlier than spring wheat (Figure 2). Even given suitable sowing opportunities, it is rare for spring wheat to finish flowering before the onset of often hot and dry conditions in June. High temperatures and water stress during the critical period of yield determination prevent spring wheat yields from rivaling those of winter wheat, and increase the risk associated with spring sowing.

As severe winter conditions prevent the sowing of spring wheat until at least late winter, and there has been little success in breeding cultivars fast enough to match the phenology of early-sown winter cultivars, winter wheat cultivars will continue to have a developmental and, very likely, a yield advantage over spring wheat in the PNW drylands. In this respect, growers have greater flexibility in Australia as similar yields and flowering in the optimal period can be obtained from both early sown winter wheat and May-sown spring wheat. In the future, the fast spring phenology of wheat cultivars developed for late sowing in southern Australia may play a role in developing faster spring cultivars to achieve timely flowering from a spring sowing date in the PNW.

### Winter Hardiness (G)

While wheat is most susceptible to cold stress during the reproductive phase, extreme cold temperatures can also damage or kill plants during vegetative stages. While winter cultivars are more suited to survival and growth in cold conditions, there is also variation within winter cultivars for winter hardiness. Cox and Shelton (1992) found that under conventional tillage across five seasons in North Dakota, the hardiest winter wheat cultivar (Norstar) had a winter survival rate of 74%, whereas other cultivars had survival rates of as low as 36%. Winter in the PNW is often harsh, with average minimum temperatures below 0°C. Crowns of winter-hardy cultivars can be held at −12°C for 15 days or longer with little or no damage (Skinner and Garland-Campbell, 2014). As little as 2–4 cm of snow cover provides the insulation necessary to trap residual soil heat and buffer against air temperature extremes that prevent killing temperatures in the crown, even during periods when air temperatures drop to −24°C. Fields with ample surface residue from the preceding crop trap snow more effectively than conventionally tilled fields (Cox et al., 1986; Papendick and McCool, 1994). In addition, the deep furrows created with deep sowing [see section “Deep Sowing (M)”] trap snow more effectively than a smooth surface.

Soil temperature at crown depth, not air temperature, is the critical factor affecting plant survival (Donaldson, 1996). Although extreme cold may kill the aboveground portion of the plant, recovery is still possible if the crown is alive. The most winter hardy PNW cultivars set crowns as deep as 3 cm below the soil surface (Donaldson, 1996).

Cold hardiness is not a static condition, but rather changes with time, temperature, and soil moisture status. Winter wheat is at most risk of winterkill when extreme cold is preceded by warm or mild conditions. Winterkill events in PNW mostly occur when cold air masses move south from Canada and are frequently associated with winds of 11 m/s (40 km/h) or greater. Wheat plants without snow cover desiccate under such cold and wind. Without snow cover, even fully hardened plants generally cannot withstand air temperatures of −23°C for more than 10 h. Severe winterkill events in the PNW drylands, which cause total crop loss, occur on average about once every 15 years.

Winter conditions in southern Australia are much milder than those of the PNW (Figure 2), and both winter and spring wheat cultivars are suitable for sowing in autumn. The increase in frequency and severity of frosts in southern Australia during winter and spring in recent decades has coincided with the recent trend in earlier sowing. As a result of these management and climatic changes, growers frequently experience losses due to frosts that occur early in the reproductive phase (stem elongation) which are referred to as stem frosts. Cold temperatures can cause pre-heading stem damage if air temperatures approach <−6°C, and if the spike emerges after such a frost event, this damage often presents as a bleached section with incomplete spike structure and aborted florets as explained in Frederiks et al. (2015). However, the increased incidence of stem elongation frost damage in southern Australia favors winter cultivars similar to the winterkill conditions commonly experienced in the PNW.

Reproductive frost induced sterility is best described in Martino and Abbate (2019). Previous recommendations for growers have been to delay sowing of spring wheat cultivars to ensure crops flower after the high frost risk period in early spring, despite reducing length of crop lifecycle and yield potential. Genotypic frost tolerance of cultivars can therefore be synergistic with crop management, as it allows cultivars to be sown earlier without encountering unacceptable frost risk; modeling conducted by An-Vo et al. (2018) showed that improving frost threshold temperature from 0 to −1°C would move the optimum sowing date 35 days earlier at a site in semi-arid southern Australia. The availability of suitable winter wheat cultivars has enabled growers in frost prone environments to capture early sowing opportunities, achieving stable flowering time, increased yield potential and avoidance of stem frost. The incorporation of winter wheat in the sowing program is commonly used as a frost mitigation tool in southern Australia.

### Early Sowing (M)

Sowing date is one of the most important management practices determining yield in low-rainfall environments (Bodner et al., 2015), and in combination with cultivar selection is one of the few options available to growers to reduce the risk of frost, heat and drought damage. Late sown wheat not only limits the amount of biomass wheat can accumulate, but also increases the risk of drought and exposure to high temperatures during the reproductive period.

For the PNW, if satisfactory winter wheat stands cannot be achieved from deep sowing in late August-early September, growers will wait and sow at a shallow depth of 2–3 cm into dry soil (referred to as ‘dusting in’) around mid-October and wait for the onset of fall rains. Such late sowing reduces winter wheat grain yields by 35–40% compared to early-sown winter wheat in east-central Washington (Higginbotham et al., 2011, 2013). If fall rains do not arrive until late November, grain yield potential of late-sown winter wheat will likely be reduced by 50% or more compared with early-sown winter wheat (Schillinger, 2016). Delayed sowing is not nearly as detrimental to grain yield potential in north-central Oregon (Bolton, 1983; Machado et al., 2015), where temperatures are warmer in the fall, winter, and spring.

The reduced reliability of autumn breaking rains in southern Australia has delayed the earliest sowing opportunity within the traditional sowing window, at a cost to biomass development and protection from heat and drought stresses. The practice of ‘dry sowing’ (the antipodean equivalent of dusting in, see Fletcher et al., 2015) has emerged partly in response to these reduced establishment opportunities. Seeds are sown into a dry seedbed on a set date, rather than in response to rainfall. Dry sowing on a fixed date has been shown to increase farm-level production without increasing production risk (Fletcher et al., 2015). This practice is complementary to the early sowing of winter cultivars in improving overall timeliness of flowering on farms (Hunt et al., 2019b).

Kirkegaard and Hunt (2010) used simulation to compare modern wheat management practices with a conventional tillage system at a site near Kerang in the Victorian Mallee from 1969 to 2009. Moving the start of the sowing period from late May to late April increased wheat yield by 467 kg/ha (30%) compared to the conventional system; when combined with complementary practices of stubble retention, control of summer fallow weeds and rotation with a forage pea crop, the total yield increase was 2,403 kg/ha.

The flexibility of growers in these regions to adapt sowing dates in response to early rainfall is currently constrained by a lack of suitable winter cultivars. When early sowing is combined with current high-yielding spring cultivars, flowering often occurs earlier than the OFP for a particular region (Flohr et al., 2018). This reduces average yield by exposing wheat to frost during the flowering period. Early sowing needs to be combined with suitable cultivars with an obligate vernalization requirement (i.e., wheat with high flowering date stability discussed above).

## Declining Water-Limited Yield Potential (E)

In a well-managed production environment free from other constraints, modern elite wheat cultivars have a maximum water productivity of 2.5 kg of grain per cubic meter of transpired water (25 kg ha^–1^ mm^–1^) (Sadras and Lawson, 2013). This places an upper limit on yield based on the available water supply during a cropping season (French and Schultz, 1984; Sadras and Angus, 2006). Water-limited potential yield (PY_W_) therefore refers to the maximal attainable yield when only water limits crop production, and optimal cultivars and agronomy practices are used, and abiotic and biotic stresses are minimized (Fischer, 2015). Water supply therefore places an upper limit on yield in semi-arid environments.

As mentioned above, growing season rainfall in southern Australia has declined in recent years, contributing to a 27% decrease in PY_W_ since 1990 (Hochman et al., 2017). Top growers have been able to maintain yields using modern cultivars and better management but are now approaching the most economically efficient yield (van Rees et al., 2014, 2015; Hochman et al., 2017). For these growers, future advances in yield will need to come from an increase in PY_W_, either through the extension of the growing season to capture more precipitation, an increase in stored soil water at the beginning of the season, and/or the release of new cultivars with a higher transpiration efficiency for grain than currently available cultivars. The development of winter wheat cultivars adapted to semi-arid cropping regions has the potential to achieve these goals when managed correctly, thus increasing yields above current limits (Hochman and Horan, 2018).

Although potential wheat yields are predicted to increase in the PNW due to anthropogenic climate change (Karimi et al., 2017; Stöckle et al., 2018), PY_W_ has traditionally been lower in the PNW compared to southern Australia, and the synergies offered by early-sown winter-type wheat have been used in the PNW for over a century to increase yield potential compared to later-sown wheat.

### Increased Biomass Compared to Spring Wheat (G × M)

Lengthening crop life cycle is one of the simplest ways to improve crop yield potential through increased biomass and grain number. Due to their vernalization requirement, winter cultivars spend a longer time period in the vegetative phase compared to spring cultivars. This means more leaves and potential tillering sites are initiated and early-sown winter wheat accumulates more vegetative biomass than spring wheat sown at the optimal time. Grain yield and grain number potential is predominantly determined by biomass accumulation during the critical growth period prior to anthesis (Fischer, 1985), while grain weight is a function of water use and temperature immediately prior, during and post anthesis (Savin et al., 1999; Plaut et al., 2004). The greater biomass accumulated by winter wheat cultivars is theoretically an advantage over spring wheat, especially those sown late.

Experiments conducted in Australia by Gomez-Macpherson and Richards (1995) and in reviewed experiments of others (Batten and Khan, 1987; Connor et al., 1992) found that grain yields of slow developing cultivars were equivalent to faster developing cultivars sown later despite similar or greater biomass in early sown cultivars due to a lower harvest index. More recent results in south-eastern Australia have demonstrated that the grain yield of slow developing wheat sown early has been equivalent to that of faster developing cultivars with a similar flowering time but with a lower harvest index (Flohr et al., 2020).

Similarly, in the PNW, early sowing always increases straw production, with August sowing dates more than doubling straw produced from the later (October) sowing in all years (Donaldson et al., 2001). In these environments the harvest index (HI) is inversely related to straw production, which means that early sowing always results in the lowest HI; similar to Australian production systems. In the PNW, early sowing of winter wheat is associated with a high number of spikes per unit area, higher grain weight, but lower number of grains per spike (Thill et al., 1978). More recent studies by Donaldson et al. (2001) found that spikes per unit area were consistently higher from earlier planting dates resulting in higher grain yields as there were limited compensatory trade-offs in grain weight and grain per spike.

It has been possible in the PNW to increase both straw yield and grain yield. Improving biomass should be viewed as an opportunity for winter wheat production in semi-arid environments. While it was possible in the PNW to further increase straw production in tall standard-height cultivars this also resulted in lower HI and grain yield (Donaldson et al., 2001) similar to the experiences currently being observed in Australia. A likely explanation is that increased plant height and more leaves lead to competition for carbohydrates between the developing spike and elongating stem of early sown crops (Gomez-Macpherson and Richards, 1995). Genetic solutions such as the development of winter wheat cultivars that can maintain improved biomass production from earlier sowing and more effectively partition accumulated biomass into grain yield will be able to maximize the synergy between early sowing and biomass accumulation, increasing grain yield above current standards (Porker et al., 2020).

#### Increasing Harvest Index (G × M)

In the PNW, increased straw production is considered advantageous for erosion control and for enhancing the capture of winter precipitation in the soil. Management solutions such as sowing rate have been proposed in both PNW and Australia as a strategy to increase straw production, HI, and yield.

Donaldson et al. (2001) at Lind, WA investigated the effect of sowing rate on straw production and HI. Medium (135 seeds m^2^) to high (195 seeds m^2^) seeding rates were favored for increased straw production. Lower sowing rates (65 seeds m^2^) increased HI and achieved similar yields to higher seeding rates but this reduced straw production and lowered spikes per unit area. Therefore, lower seeding densities (<70 plants m^2^) were not recommended for the PNW.

In Australia, reducing plant density has also been proposed as a way of reducing early DM accumulation and improving HI in early established slow developing cultivars for improved grain yield (Hunt et al., 2012; Kirkegaard et al., 2014). However, few studies have demonstrated an advantage from reducing plant density in early sown winter wheat in Australia, and effects have been generally small (Porker et al., 2020). Sowing rate responses appear much larger in the PNW relative to Australia. In the PNW the effects of sowing rate on plant growth and development were so large they masked any differences in cultivars responding differently (Donaldson et al., 2001). In Australia, higher seeding densities are favored in early sown winter wheat for dual purpose use (used for early season forage), and in situations where greater weed competition is required.

The long vegetative phase of slow developing wheat also suggests that deferring nitrogen inputs until after the start of stem elongation could significantly increase yield. Few published experiments have reported a consistent effect of N fertilizer timing on crop yield in winter cultivars under Australian conditions from early sowing dates due to other confounding factors such as high residual N and stem frost (Porker et al., 2020), though studies in other Mediterranean-type environments suggest a benefit to grain yield and protein through splitting N application between sowing, GS15 and GS30 (Ercoli et al., 2013).

### Increased Rooting Depth Compared to Spring Wheat (G × M)

As root length and mass is influenced by sowing date and phase duration (Barraclough and Leigh, 1984; Kirkegaard et al., 2015), early-sown winter wheat has more and longer roots compared to later sown spring wheat (Entz et al., 1992; Kirkegaard and Lilley, 2007; Williams et al., 2013). Although the roots of winter and spring cultivars penetrate soil at a similar rate, the longer period of root proliferation increases subsoil specific root length and maximum rooting depth in early-sown winter cultivars (Entz et al., 1992; Kirkegaard and Lilley, 2007; Thorup-Kristensen et al., 2009). Maximum rooting depth is highly correlated to the maximum depth from which water is extracted (Entz et al., 1992). Kirkegaard and Lilley (2007) suggested that this advantage of winter wheat is only pronounced in years with sufficient precipitation to wet the entire soil profile. Flohr et al. (2020) demonstrated this experimentally in slow developing spring cultivars, with the added condition that low rainfall is required during the critical period to force reliance on deep stored water.

In the PNW, winter wheat effectively extracts soil water to a depth of 150 cm or more. This increased rooting depth allows the crop to access more water during anthesis and grain fill, during which time water use efficiency of subsoil water averages 35 kg/ha.mm, compared to a water use efficiency of 20–25 kg/ha.mm across the entire growing season (Kirkegaard et al., 2007; Flohr et al., 2020). During grain fill, water in the surface 100 cm has often been depleted and there is little likelihood of any substantial rain. Entz et al. (1992) found that winter wheat had access to more total water at anthesis in seasons with dry finishes, whereas when significant rainfall occurred late in the growing season, post-anthesis water availability was similar in spring and winter cultivars.

## Reduced Establishment Opportunities (E)

If autumn rainfall continues to decline, southern Australian growers will regularly need to be able to establish wheat outside the traditional sowing window. Sufficient rainfall to create early sowing opportunities does not occur in every year, and the probability of receiving an early sowing opportunity varies among locations across southern Australia. The opportunity to establish wheat independently of rainfall offers growers the ability to extend the establishment window and creates flexibility in crop selection. There are whole-farm benefits to establishing wheat in the absence of rainfall, as the remaining crops can be sown in a timelier fashion.

Establishing wheat on stored soil water is common practice in the PNW and has been for many decades. Deep sowing may in future years become practiced in semi-arid southern Australia, where growers have the opportunity to learn from the advancements in genotype, sowing machinery and farm management that have made this technique possible in the PNW (Hunt et al., 2019a).

### Deep Sowing (M)

There is a fundamental farming system synergy between 13-month fallows, winter wheat, and deep sowing in the drylands of the PNW. Very little rain falls from July to September and what does fall generally evaporates from the soil surface within a few days. As described above, winter wheat established in October (after 15 months of fallow) or November when the surface has been wetted by rain suffers a large yield penalty in comparison to stands established into stored soil moisture in late summer. Planting back-to-back winter wheat (i.e., without the long fallow) is a recipe for disaster (Schillinger, 2016) that no dryland growers practice.

**FIGURE 4:**
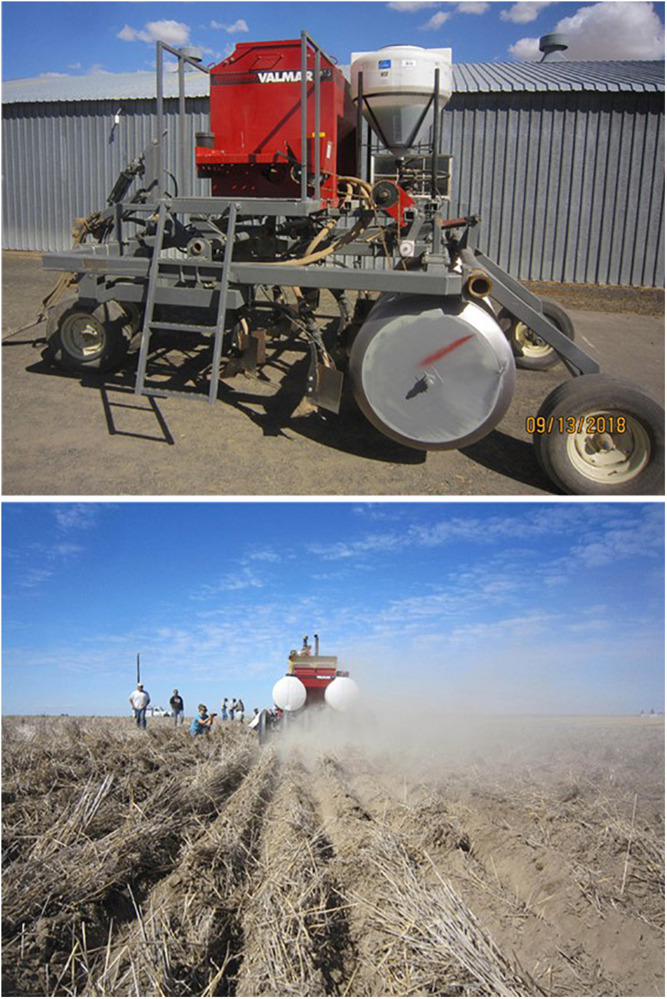
A prototype deep-furrow drill fabricated at the Washington State University Lind Dryland Research Station. Commercially available deep-furrow drills cannot pass through heavy surface residue without plugging and are not sturdy enough for seed openers to penetrate through the hard, dry surface of no-till summer fallow. Growers and scientists seek a dual-purpose drill to sow winter wheat into heavy residue in both tilled and no-till fallow conditions. Hoe-type openers of the drill must be able to place seed as deep as 20 cm below the surface to reach adequate soil moisture for germination and emergence. The purpose of the deep furrow is to reduce the thickness of soil covering the seed to enhance seedling emergence. Photos by W. F. Schillinger.

The system practiced by PNW dryland growers is deep sowing of winter wheat into stored fallow moisture using deep furrow drills (Figure 4). This enables growers to achieve optimal establishment times for winter wheat without having to rely on rainfall to wet seed beds. Pacific Northwest winter wheat cultivars can germinate at water potentials as low as −1.25 MPa (Wuest and Lutcher, 2013; Wuest, 2018), but a minimum water potential of −0.55 to −0.65 MPa is generally required for winter wheat seedling emergence through more than 12 cm of soil cover (Lindstrom et al., 1976; Schillinger et al., 1998). Due to thick soil cover over the seed, it is not the coleoptile that emerges from the soil but rather the first leaf after pushing through the tip of the coleoptile. The first leaf is thin and spindly and, since most often emerging under low soil water potential, lacks much emergence force or lifting capacity (Lutcher et al., 2019). The drier the soil in the seed zone and the deeper the soil cover, the longer it takes seedlings to emerge (Lindstrom et al., 1976).

The ability to emerge from depth is an essential trait for winter wheat adapted to the PNW drylands, and there has been strong selection within winter wheat breeding programs for this trait. In the Washington State University (WSU) winter wheat breeding program, F3 plots from crosses targeting the drylands are deep sown at the Lind Dryland Research Station and selections made from these. In this way, emergence from depth is selected before any other trait, including yield.

In contrast, in southern Australia deep sowing has been limited in practice until recent times. Following autumn rainfall decline, inadequate moisture at ideal sowing depth has led to growers sowing deeper to ‘moisture-seek’ (placing seed into moist soil below a layer of dry soil) to make use of residual moisture stored from summer rains or an 18-month long fallow. Their ability to do this is currently restricted by the availability of sowing equipment capable of placing seeds into moist soil at depth, and the ability of plants to emerge from depth.

### Ability to Emerge From Depth (G)

Coleoptile length is an important trait determining the ability of seeds to emerge from depth and has been studied in Australia (Rebetzke et al., 2007; Bovill et al., 2019). In the PNW, all current soft white winter wheat (SWW) cultivars are semi-dwarfs that carry emergence-impeding Rht1 or Rht2 dwarfing genes but developing standard height (i.e., no dwarfing genes) SWW cultivars is a high priority of the WSU winter wheat breeding program. Standard height hard red winter wheat cultivars are available to growers in the very dry (150–220 mm annual precipitation) areas of south-central Washington.

The long outdated standard height SWW cultivar Moro (Rohde, 1966) had a coleoptile length of 90 mm and had excellent emergence from deep sowing depths. Moro was the number one winter wheat planted by dryland growers in east-central Washington for more than 20 years (Schillinger and Papendick, 2008). In 12 deep-sowing experiments, Moro always emerged faster and better with 11–16 cm of soil covering the seed compared to semi-dwarf SWW cultivars (Schillinger et al., 1998). In addition to a long coleoptile, Moro had a first leaf length of 207 mm compared to an average of only 130 mm first leaf length for other SWW cultivars (Schillinger et al., 1998). Mohan et al. (2013) conducted a comprehensive deep-sowing emergence field trial at Lind, WA with 662 wheat cultivars collected from around the world. These cultivars had coleoptile lengths ranging from 34 to 114 mm; a length of 90 mm was ideal as emergence declined for cultivars with coleoptiles longer than 90 mm.

Modern Australian semi-dwarf wheat and barley cultivars show poor emergence when sown deep (greater than 8 cm) due to shortened coleoptiles (Rebetzke et al., 2007). Warmer soils in the future may further exacerbate poor establishment with deeper sowing (Rebetzke et al., 2016).

Pre-experimental modeling indicates substantial benefits for crop yield in southern Australia if machinery and cultivars could be developed that allowed placement and emergence of seed at depth (Kirkegaard and Hunt, 2010; Flohr et al., 2018). Rebetzke et al. (2016) have argued the case for Australian breeders to use novel dwarfing genes, such as Rht8, that do not suppress coleoptile length.

### Long Fallow (M)

#### Inland Pacific Northwest

Late summer or early autumn planting of winter wheat requires sufficient stored soil moisture to ensure establishment of a crop in the absence of precipitation. In the PNW drylands, winter wheat is grown in rotation with 13-month fallow to accumulate moisture. On average, 65% of overwinter precipitation is stored in the soil but, due to high evaporation during the dry summer months, only an average of 30% of precipitation that occurs during the 13-month fallow is stored in the soil by late August.

Following harvest in late July or early August, winter wheat stubble is generally left standing and undisturbed until at least April, using a non-selective herbicide in late winter or early spring to control weeds. In mid-April or later most growers till fallow ground with an undercutter sweep implement or field cultivator to a depth of 10–13 cm to break soil capillary pores, creating a dry surface soil mulch to slow the upward flow of water and to thermally reduce heat flow into the soil. In the hot and dry summer months, the transfer of heat from the surface to water below is a primary driver for evaporation. Liquid water will move upward to the depth of tillage where thereafter it moves through the dry soil mulch via vapor flow. Near-surface soil water loss with no-till fallow is generally greater than with tilled fallow during the dry summer and the drying front with no-till fallow often moves below the depth that can be reached with deep-furrow drills. The physics of soil water dynamics in tilled versus no-till fallow in the PNW drylands has been reported in dozens of journal articles in the past 100 years and is well summarized by Hammel et al. (1981) and Papendick (2004).

A huge downside to tilled fallow is the risk of wind erosion, which is a major soil and air quality concern in the PNW drylands. In recent decades, most growers take care to leave ample surface residue and maintain a cloddy surface during fallow, but dust storms are still frequent, especially when straw production (and yield) of the preceding crop was low. No-till fallow is ideal for wind erosion control and acreage of no-till fallow is increasing. For example, in the milder climate of north-central Oregon where early October is an optimum sowing date (Bolton, 1983), more than 75,000 hectares of no-till summer fallow is practiced (Machado et al., 2015). No-till fallow is also practiced in the <200 mm average annual precipitation of south central Washington where storing adequate water during fallow for early sowing is not achievable most years. Additionally, no-till fallow is becoming increasingly popular in the relatively cool climate of Douglas County in north central Washington where the glacial soils farmed there are only 45–90 cm deep. However, tillage-based fallow is still the most common practice in the PNW drylands due to the seed-zone water phenomena discussed above.

Two back-to-back years of fallow is infrequently practiced in the PNW during drought periods when soil water accumulation during the first fallow year is poor. This extended fallow is called “double fallow” as it captures two winters of precipitation. With double fallow spring wheat is sown after a 20-month fallow or winter wheat after a 25-month fallow. A double fallow is inherently inefficient because: (i) the percentage of precipitation captured in the soil during the second winter is much lower than during the first winter, and (ii) spring wheat sown after an 18-month fallow will generally have significantly lower grain yield that winter wheat planted after a 13-month fallow (Young et al., 2015).

#### Semi-Arid Southern Australia

Sowing spring wheat in late autumn following an 18-month fallow was a widespread practice in semi-arid southern Australia until the mid-1980s (Ridge, 1986), but in recent decades cereal crops have been more commonly grown in rotation with profitable break crops such as canola or pulses. In these continuous cropping systems, an annual 5-month fallow is maintained from the harvest of one crop in early summer to the sowing of the next in late autumn (Hunt and Kirkegaard, 2011).

An 18-month fallow generally increases the yield of wheat compared to wheat grown following another wheat crop. This fallow is functionally equivalent to the 13-month fallow found in the PNW, as a field is left out of production for one growing season while precipitation from one winter is stored in the soil. The yield advantage of wheat grown on an 18-month fallow is higher in fields with a high plant-available water capacity, and the relative benefit of the practice is therefore more advantageous in south-eastern Australia than the sandier western cropping regions (Oliver et al., 2010). Unlike equivalent sequences in the PNW, total production is usually higher in continuous cropping systems than wheat-fallow rotations (Angus et al., 2015), but there are several whole-farm benefits, including reduced input costs, increased timeliness of sowing, and decreased income risk that increase the favorability of including a strategic 18-month fallow in crop sequences (Cann et al., 2020).

These yield and management advantages also work in synergy with the yield and management advantages offered by winter wheat. Flohr B. et al. (2018) simulated twelve different genotype × management strategies for spring and winter wheat production at three low-rainfall zones in southern Australia from 1997 to 2016. The highest yielding strategy at all three sites was a long coleoptile winter wheat grown following a long fallow. At Walpeup, in the Victorian Mallee, this strategy achieved a higher simulated yield than continuous production of both a short coleoptile spring wheat variety and a long coleoptile winter wheat in 16 of 20 years. The average yield of the long coleoptile winter wheat cultivar increased from 3.2 to 4.5 t/ha when rotated with long fallow as opposed to continuous wheat production; a relative yield increase of 1.3 t/ha. On the other hand, the yield of a continuously cropped long coleoptile fast spring wheat increased from 2.7 to 3.6 t/ha when rotated with long fallow; a relative yield increase of only 0.9 t/ha. This demonstrates that the adoption of multiple genotypic and management practices can have an additive effect, and that the yield of winter wheat is maximized when G × M is optimized.

## Increasing Business Costs and Risk

While in recent years, the top growers of southern Australia have successfully reduced the gap between on-farm yields and PY_W_ to an economically optimal level (van Rees et al., 2014; Hochman et al., 2017), whole-farm profit margins have stagnated. While farm incomes in semi-arid southern Australia increased by 82% from 1994–1998 to 2008–2012, total costs increased by 89% (van Rees et al., 2014, 2015). Income volatility has also increased. In Victoria, wheat revenue variance was greater for 1992 to 2009 (0.38 – linear area trend) than 1973 to 1991 (0.14) or 1955 to 1975 (0.15) (Kingwell, 2011). Growers in semi-arid Western and South Australia, where 85–95% of grain is exported, are also further exposed to the volatility of international markets (Laurie et al., 2018). Increasing profit in the future will require not only the release of cultivars with higher water-use efficiency and therefore increased yield potential, but also potential changes at the system or whole-farm level – including farming more land and diversifying through the inclusion of livestock.

Although these management strategies have the potential to increase whole-farm profit, they also carry increased risk – increasing cropped area increases the time needed for seeding to achieve timely sowing, while incorporating livestock into the enterprise requires supplementary feeding during times of low pasture availability (predominantly autumn in southern Australia). In this context, the development pattern of winter wheat is potentially advantageous in reducing income risk and increasing whole-farm yield.

In the PNW, increasing costs have until recently been offset by increased resource-use efficiency of cultivars, machinery and fertilizers. However, increased investment in land area is also necessary to maintain or improve profit margins (Schillinger and Papendick, 2008). Income risk has increased for PNW growers with increasing year-to-year variability of grain price since 1975. However, one core difference between the economies of southern Australian and PNW cropping enterprises is the role of the government. Compared to Australian growers, PNW growers have greater access to government-sponsored subsidies and incentives (Barnard et al., 1997), such as subsidized multi-peril crop insurance available for wheat, canola, dry pea, and triticale. The Conservation Reserve Program provides eligible landowners 10-year paid contracts to plant perennial grasses and shrubs in lieu of cropping. The Environmental Quality Incentives Program, managed at the local (County) level, provides incentives to adopt environment-friendly cropping practices such as direct seeding into no-till fallow. Such federal government programs reduce risk, help stabilize farm income, and buoy cropland values; albeit at considerable expense to US taxpayers.

### Grazing (M)

In Australian mixed-farming systems, winter cultivars are commonly sown early as dual-purpose crops, whereby they provide livestock forage during the vegetative stage, and later regrow for grain production (Virgona et al., 2006; Harrison et al., 2011). When sown early, the vernalization response of winter cultivars results in an extended vegetative phase, which provides a grazing opportunity during autumn and winter when comparative growth rates of other pastures are slow (Virgona et al., 2006). Additionally, grazed crops are able to recover to achieve similar yields to non-grazed crops (Dove and McMullen, 2009), increasing the net economic gains from these crops by 25–75% (Bell et al., 2014). Other farming system benefits of dual-purpose crops that have been reported include reduction in crop height, risk of lodging and post-harvest stubble loads (Harrison et al., 2011). Flexibility in sowing time and delayed phenological development after grazing can reduce risk of frost damage in frost-prone environments (Porker et al., 2020), as well as enabling spelling of pasture paddocks during autumn and winter, increasing whole-farm feed supply and production (Bell et al., 2015).

Cattle grazing of winter wheat during its vegetative stage of development during the fall is also possible in the PNW with no reduction in subsequent grain yield compared to non-grazed wheat. However, grazing is practiced on only a small scale. Farms today are large and specialized on crop production; most PNW dryland wheat growers do not raise cattle.

### Whole-Farm Timeliness (G × M)

Average farm size in southern Australia and the PNW, both in corporate and family owned farms, has increased steadily for several decades (Anderson et al., 2016). Timely sowing is required to maintain whole-farm yield (Fletcher et al., 2015). Increased farm size requires increased investment in machinery in order to sow a crop in timely fashion. Alternatively, in southern Australia sowing winter wheat when favorable sowing opportunities arise allows growers to expand the sowing window and increase timeliness of sowing at the whole-farm level (Fletcher et al., 2019; Hunt et al., 2019b). As farm size continues to increase, the opportunity to extend the sowing window by growing early-sown winter wheat will continue to grow in appeal as a mode of maintaining whole-farm yield.

In the PNW drylands, where winter wheat after a 13-month fallow dominates, the optimum sowing window is only 2–3 weeks. It is common for growers to have two or more tractor-drill units sowing concurrently and/or conduct their sowing operations 16–24 h per day 7 days a week. Sowing winter wheat during this time window is, by far, the most time-critical and important (and stressful) field operation for PNW dryland wheat growers.

## Potential Barriers to Uptake and Implementation

Winter wheat production in semi-arid southern Australia is currently in its early stages, with winter cultivars sown only by those considered “innovators” or “early adopters” by Rogers’ diffusion of innovations theory (Rogers, 2010). While the grower population in semi-arid southern Australia has historically been responsive to new technology and management practices (D’Emden and Llewellyn, 2006; Llewellyn et al., 2012; Fletcher et al., 2016), there are a number of key obstacles that remain in the way of the widespread early sowing of winter wheat cultivars in the region.

### Lack of Suitable Cultivars

Following a wave of privatization in the early 2000s, Australian breeding programs and therefore objectives have almost exclusively been dominated by private breeding companies (Lindner, 2004). The release of wheat cultivars in Australia is therefore tied to profitability for breeding companies (through end-point royalties), rather than increasing sowing flexibility. Spring wheat cultivars, which have greater adaptation across environments compared to winter wheat lines (Porker et al., 2019), have therefore dominated cultivar releases in recent decades.

Despite the above, Australian breeders have recently reacted to growing interest from dryland growers for suitable winter wheat cultivars. Longsword, a fast winter wheat designed for semi-arid environments, was released in 2018 by Australian Grain Technologies (AGT) as a feed-quality cultivar (Flohr et al., 2018). AGT have also established a dedicated winter wheat breeding program at Wagga Wagga in southern Australia, and the first cultivar from this program (Illabo) was released in 2018. Several other breeding companies have also signposted upcoming releases of winter lines. While early innovators in semi-arid environments have taken to sowing winter wheat lines including Longsword, early-sown winter wheat will not be grown by a majority of growers until cultivars are released that are suited to the growing environment and can meet the requisite milling quality checks to maximize profit margin. Similarly, sowing of winter cultivars into stored subsoil moisture will not be possible until winter varieties with the ability to emerge from depth have been released. Future winter wheat breeding programs will therefore have to address not only current grower preferences, but also identify and fulfill emerging and future opportunities for G × M synergies to increase yield.

Specialized wheat breeding programs have been ongoing in the PNW since 1894 (Schillinger and Papendick, 2008). Unlike the Australian system, state land-grant university breeding programs are common in the United States and have been responsible for the release of hundreds of wheat cultivars. Wheat cultivars released in recent years by university breeding programs are no longer “public” cultivars (i.e., no royalty fee), but rather growers must pay a seed royalty fee to the university and are not allowed to store their harvested grain for seed. This has resulted in head-to-head competition with private wheat breeding companies who have developed many excellent lines. Winter cultivars bred for the PNW are unlikely to be suitable for Australian growers due to target markets for grain, region-specific fungal and nematode pathogens and the development speed of cultivars, which are still likely to be too slow to ensure flowering within the narrow OFPs in southern Australia. Winter wheat breeding programs will need to be specific to Australian climates to ensure that released cultivars have a stable flowering time (through vernalization) but also a fast development rate to ensure flowering is completed before the onset of hot, dry conditions, a requirement that will grow in importance as average temperatures continue to rise.

#### Environmental Specificity of Cultivars

Australian growers will require greater diversity in winter wheat for a range of flowering environments compared to spring cultivars. Although the optimal flowering period is different from region to region even within low-rainfall cropping zones, growers across these regions have traditionally manipulated sowing date of high yielding spring cultivars to ensure they flower during the OFP. Due to the stable flowering time of winter cultivars, adaptation will be driven by cultivar flowering time and coincidence with optimal flowering periods in the different environments (Hunt et al., 2019b; Porker et al., 2019). A change in development pattern (genotype) rather than sowing time (management) will be required for each different flowering time environment. This means breeding programs and growers will need to target development patterns to suit different flowering environments – fast for warm, low rainfall environments and mid to mid slow for cool medium rainfall environments. This limits the potential profitability of winter wheat lines for plant breeders, who need to invest the same (if not more) resources into winter wheat candidates for specific regions as they do for spring lines which can be grown across a wider range of environments. As released winter wheat cultivars are only likely to flower within the optimal flowering period and therefore achieve optimal yield across a smaller range of environments, their uptake is already restricted by their phenology.

There have been several suggested solutions for this problem. For environments with a narrow OFP, where frost can cause major yield reductions, sowing a slower winter wheat with heat and drought tolerance can ensure firstly that reproductive frost events are avoided by late flowering, and secondly that the impact of drought and heat events are minimized (Hunt et al., 2019a). Such cultivars would have more widespread geographic adaptability than other winter wheat cultivars, as they would be suitable for sowing not only in environments where they flower during the OFP, but also environments where they flower “too late,” as long as their heat and drought tolerance prevents large yield reductions from stress events. Recent research has also suggested that the phenology of some wheat cultivars can be “reset” through induced vegetative stress or heavy grazing. This may extend the use of some winter wheat cultivars, particularly those suitable for use as dual-purpose wheat, but more research is needed to explain how management can be used to manipulate the phenology of winter wheat.

### Implications for Agronomic and Whole-Farm Management

While some whole-farm factors such as timeliness of sowing favor the inclusion of early-sown winter wheat into farming systems, there remain several considerations that need to be accounted for when including winter wheat in a cropping program. One major traditional impediment to early sowing has been weed control. Historically, growers would need to wait until weeds had emerged in mid-autumn to control them, using either tillage or more recently knockdown herbicides (glyphosate and paraquat). In both southern Australia and the PNW, the advent of pre-emergent herbicides that can be applied before sowing means that growers no longer need to wait until weeds have emerged to begin control methods.

Additionally, while winter wheat cultivars offer increased sowing time flexibility, growers also need to have seed on hand in preparation for sowing. This becomes increasingly problematic in environments where early sowing opportunities are unreliable, as harvested seed needs to be held on farm in readiness for an early sowing opportunity that may not eventuate for several years. However, the stable flowering time and yield advantage of winter wheat potentially outweighs the additional inconvenience of storing winter wheat seed on farm. This problem is not encountered in the PNW due to the widespread availability of winter cultivars.

A transition to a system in which winter wheat is established on stored soil water rather than rainfall will also require the contemporaneous development of suitable cultivars, machinery and management skill. In the PNW, the deep placement of winter wheat seed is only suitable for cultivars that can emerge through 10–15 cm of soil cover (Schillinger et al., 2017). The development of deep-furrow split-packer drills that allowed this deep placement in the mid-1960s occurred almost simultaneously with the release of the cultivar Moro, a cultivar with excellent emergence from depth. Availability of similar machinery in a southern Australian environment would not only require significant whole-farm expenditure from growers, but also need to be accompanied by high-yielding cultivars with excellent seedling emergence in order to facilitate widespread uptake. Access to PNW machinery and winter wheat germplasm would likely accelerate this process if emergence traits were to be incorporated into southern Australian breeding objectives. It would also be imperative that deep sowing into fallow moisture fit into the no-till and stubble retained farming systems which now dominate in southern Australia and have been extremely effective at reducing wind and water erosion and delivered many production benefits. The machinery challenges of sowing into uncultivated heavy soil types common in much of southern Australia would likely be greater than into the sandy silt loam soils of the PNW drylands.

## Conclusion

The history of winter wheat production across the inland Pacific Northwest of the United States provides insight into how early-sown winter wheat cultivars can be established in the absence of precipitation, flower during the optimal flowering period and significantly out-yield later-sown spring wheat genotypes. As cropping conditions in southern Australia begin to converge with those of the PNW, Australian growers have the opportunity to learn from both the successes and the challenges of winter wheat production in the PNW, and adopt the most advantageous components whilst still exploiting the benefits of conserved crop residues and crop rotations. The yield advantage of winter wheat in the PNW has been the result of several genotype × management synergies, some of which are inherent to winter genotypes, but others which have been developed in response to the nature of the environment in which they are grown. Future semi-arid southern Australian cropping systems may therefore be able to increase flexibility by sowing winter wheat cultivars after early rainfall, increase profitability and decrease risk by grazing dual-purpose winter wheat, and establish crops independently of rainfall by deep-sowing winter cultivars with long coleoptiles into stored soil water during late summer or early autumn.

## Author Contributions

DC conceptualized the manuscript and wrote most of the analysis pertinent to southern Australia. WS contributed sections relevant to PNW. JH, KP, and FH made substantial written contributions to sections on southern Australia. DC, WS, and JH prepared the figures. All authors reviewed and edited the manuscript.

## Conflict of Interest

The authors declare that the research was conducted in the absence of any commercial or financial relationships that could be construed as a potential conflict of interest.
